# Deposition of amyloid β in the walls of human leptomeningeal arteries in relation to perivascular drainage pathways in cerebral amyloid angiopathy^☆^

**DOI:** 10.1016/j.bbadis.2015.08.024

**Published:** 2016-05

**Authors:** Abby Keable, Kate Fenna, Ho Ming Yuen, David A. Johnston, Neil R. Smyth, Colin Smith, Rustam Al-Shahi Salman, Neshika Samarasekera, James A.R. Nicoll, Johannes Attems, Rajesh N. Kalaria, Roy O. Weller, Roxana O. Carare

**Affiliations:** a Faculty of Medicine, University of Southampton, Tremona Road, SO16 6YD, UK; b Centre for Clinical Brain Sciences, University of Edinburgh, UK; c Institute of Neuroscience, Campus for Ageing and Vitality, Newcastle University, UK

**Keywords:** Amyloid-β, Leptomeningeal arteries, Perivascular drainage, Basement membranes, Cerebral amyloid angiopathy

## Abstract

Deposition of amyloid β (Aβ) in the walls of cerebral arteries as cerebral amyloid angiopathy (CAA) suggests an age-related failure of perivascular drainage of soluble Aβ from the brain. As CAA is associated with Alzheimer's disease and with intracerebral haemorrhage, the present study determines the unique sequence of changes that occur as Aβ accumulates in artery walls. Paraffin sections of post-mortem human occipital cortex were immunostained for collagen IV, fibronectin, nidogen 2, Aβ and smooth muscle actin and the immunostaining was analysed using Image J and confocal microscopy. Results showed that nidogen 2 (entactin) increases with age and decreases in CAA. Confocal microscopy revealed stages in the progression of CAA: Aβ initially deposits in basement membranes in the tunica media, replaces first the smooth muscle cells and then the connective tissue elements to leave artery walls completely or focally replaced by Aβ. The pattern of development of CAA in the human brain suggests expansion of Aβ from the basement membranes to progressively replace all tissue elements in the artery wall. Establishing this full picture of the development of CAA is pivotal in understanding the clinical presentation of CAA and for developing therapies to prevent accumulation of Aβ in artery walls. This article is part of a Special Issue entitled: Vascular Contributions to Cognitive Impairment and Dementia edited by M. Paul Murphy, Roderick A. Corriveau and Donna M. Wilcock.

## Highlights

• Lymphatic drainage of the brain is along basement membranes in the walls of arteries.
• Perivascular lymphatic drainage fails with age and arteriosclerosis.
• Aβ deposits in the perivascular drainage pathways of leptomeningeal arteries as CAA.
• As CAA progresses, Aβ replaces all elements of the ageing artery wall.
• Facilitation of perivascular drainage may prevent CAA and delay Alzheimer's disease.

## 1 Introduction

Deposition of insoluble amyloid β (Aβ) within the extracellular spaces of the brain and the accumulation of hyperphosphorylated tau within neurons as neurofibrillary tangles are major features in the pathology of Alzheimer's disease (1). Aβ is also deposited in the brain with age in non-demented individuals in addition to those with AD, strongly suggesting that there is an age-related failure of elimination of Aβ from the brain [2], [3], [4], [5], [6]. In addition to plaques in the brain, Aβ is deposited in the walls of cerebral capillaries and arteries as cerebral amyloid angiopathy (CAA) with age and in AD. In late stages of CAA, the walls of cerebral arteries are completely replaced by Aβ and this may be associated with CAA-related intracerebral haemorrhage [7], [8], [9], [10].

Due to the close association of CAA with both Alzheimer's disease and with CAA-related intracerebral haemorrhage, the main aim of the present study is to establish the sequence by which Aβ accumulates in artery walls in the development of CAA. Despite the very infomative studies on the distribution of CAA (11) and its quantitation (12), relatively little is known about the sequence of events that leads to increasing deposition of Aβ in artery walls in CAA in the human brain (13). Establishing a full picture of the development of CAA is pivotal in understanding the clinical presentation of CAA, its detection by imaging techniques and the development of therapies to prevent the accumulation of Aβ in artery walls.

Experimental studies have shown that when tracers of equivalent molecular size to Aβ, and soluble Aβ itself, are injected into the brain, they initially diffuse through the narrow extracellular spaces of the brain but rapidly enter bulk flow pathways within the basement membranes of capillary and artery walls that represent the lymphatic drainage pathways of the brain (14). With age and possession of apolipoprotein E ε4 (apoE4), two of the major risk factors for AD, perivascular lymphatic drainage of soluble Aβ is significantly reduced [15], [16].

Impairment of perivascular lymphatic drainage appears to be related to age-related stiffening of artery walls and changes in vascular basement membranes (17). The presence of CAA is a reflection of the impaired perivascular lymphatic drainage and failure of elimination of Aβ from the brain with age and AD [6], [18], [19], [20]. Comparing confocal images of experimental tracer studies with the distribution of Aβ in vessel walls in human CAA reveals a strong correlation suggesting that CAA represents a failure of perivascular lymphatic drainage of Aβ from the ageing and Alzheimer's brain (21). In this study, we use carefully selected age-matched post-mortem brains from young and aged non-demented individuals and from patients with Alzheimer's disease to assess the stages in deposition of Aβ in the walls of cerebral arteries in the development of CAA. We also quantify the changes that occur in the immunohistochemical profile of vascular basement membrane proteins with age and AD.

## 2 Materials and methods

Sections of 10 μm thickness of postmortem human occipital cortex from the Newcastle Brain Tissue Resources and MRC Sudden Death Brain& Tissue Bank (Edinburgh) were used for this study (Table 1, Table 2). The cases were diagnosed post-mortem by JA, according to published criteria including neuritic Braak stages (22), Thal amyloid phases (23), CERAD scores (24), NIA-AA scores (25) and McKeith criteria (26) showed varying degrees of Alzheimer's disease pathology. For CAA we used recently a staging system which assesses meningeal and parenchymal CAA separately and also scores capillary CAA (see: (27)). None of the cases was diagnosed with CAA during life. The cases from the MRC Sudden Death Brain& Tissue Bank (Edinburgh) had no neurological disease during life and no significant neuropathological changes post mortem. We have excluded any cases with arteriolosclerosis/lipohyalinosis from this cohort. All samples were collected and prepared in accordance with the National Research Ethics Service approved protocols. For this study we used tissue from both young (n = 14 mean age 43.3 see Table 1) and aged (n = 20 mean age 81.45, Table 1) controls and from severe CAA cases (n = 20 mean age 82.5, Table 1). Sections were immunostained for collagen IV (Col IV, AbCam, Cambridge, UK, 1:400), nidogen 2 (polyclonal antibody produced in-house, dilution 1:1000), fibronectin (1:400, AbD Serotec, UK). A total of 1689 images were obtained of the cortical gray matter adjacent to a sulcus, using the tissue microarray feature of the Olympus Dot Slide microscope and images were analysed using Image J, for percentage area stained. Statistical analysis was performed using SPSS statistics and one-way ANOVA with LSD post hoc.

### 2.1 Triple immunofluorescence and confocal microscopy

Ten cases of CAA were diagnosed as severe CAA according to published criteria (7). Details of the antibodies used are in Table 3. The paraffin-embedded brain tissue sections were de-waxed at 60 °C for 15 min, rehydrated through graded alcohols and pre-treated with 98% formic acid at RT, 3 min. Slides were washed with 0.01 M TBS, microwaved in 400 mL 0.01 M citrate buffer (pH 6), microwaved for 25 min, and incubated with 500 μL of 15% normal goat serum and blocking medium for 30 min. Slides were incubated overnight at 4 °C with the primary antibodies (Table 3); anti-Aβ-4G8 (dilution 1:100), anti-collagen IV (dilution 1:400), and FITC-conjugated anti-SMA (dilution 1:200). Secondary antibodies used were; goat-anti-mouse IgG2b 594 (dilution 1:200) and goat-anti-rabbit 633 (dilution 1:200). To quench autofluorescence, slides were incubated in Sudan Black (1% in 70% alcohol) in the dark at room temperature, 5 min, washed with 0.01 M TBS, labelled and cover slipped with 200 μL Mowiol and Citifluor mounting medium before examination with the confocal microscope.

#### 2.1.1 Confocal microscopy imaging

The slides were viewed with a Leica TCS SP5 laser scanning confocal microscope. Leptomeningeal arteries along sulci were identified and distinguished from other vessels based on the presence of smooth muscle actin in tunica media. From each of the 10 slides, a total of 10 arteries with a diameter larger than 10 μm were imaged at × 40 objective, with a total of 100 leptomeningeal arteries imaged. In order to perform a qualitative analysis of the pattern of Aβ deposition within the leptomeningeal vessel walls, relative to the presence of SMA and Col IV single channel fluorescence confocal images were obtained in series, followed by an overlay image from both fluorescence channels. These images were viewed and analysed using Leica LAS AF 3.x windows7 software.

Image J version Fiji windows 64 software (http://fiji.sc/Fiji; NIH, Bethesda, USA) was used for the quantitative analysis of the 100 vessels to:

1. Calculate the percentage (%) of Aβ, SMA and Col IV within the total area of leptomeningeal artery wall;
2. Calculate the internal diameter of the 100 leptomeningeal arteries, as measured at its smallest point;
3. Calculate the percentage (%) of co-localisation of Aβ, SMA and Col IV within the leptomeningeal artery wall. Leptomeningeal arteries have a thicker wall, larger internal diameter and are ideal for analysis by confocal microscopy.

Using the Image J region of interest manager, the perimeter of each vessel and luminal area for the 100 vessels was traced by hand and the area of the vessel wall calculated (total vessel area − lumen area = area of the vessel wall). After calibrating each image to a known diameter the blood vessel diameter was calculated using the Image J measurement tool, using the smallest cross sectional diameter of the blood vessel as the most accurate form of measurement. In order to ensure the validation of fluorescent intensity so that only genuine fluorescence was detected, fluorescence thresholds were set for each of the colour channels: red for Aβ, green for SMA and blue for Col IV. An initial validation analysis was carried out on mock images to ensure correct judgement on the capture of genuine fluorescence. Co-localization of each fluorescence colour was calculated using colour thresholds selected in pairs to calculate the number of pixels with overlap of two colour channels; red/blue, red/green and green/blue. The Image J particle analyser tool was used to quantify the area covered by each fluorescence colour and each pair of colours. The individual fluorescence values were calculated as a percentage of total vessel wall area. This data was processed using the commercial software package MATLAB (MATLAB 6.1, The MathWorks Inc. Natick, MA, 2000) to present the percentage of total fluorescently labelled vessel wall comprised of each protein of interest as a 3D scatter plot. Relationships between the percentage of each protein within the total vessel wall relative to blood vessel diameter, correlation between amount of each protein present and patterns of co-localisation were analysed using Microsoft Excel (Microsoft Office 2010) and presented as 2D data plots. The amount of each protein present relative to the blood vessel diameter and the degree of co-localisation between the different protein pairings (Col IV/SMA, Col IV/Aβ, Aβ/SMA) were statistically analysed using Graph Pad Prism 6.0 (Graph Pad Prism inc. USA) in one-way and two-way analysis of variance (ANOVA). A P-value of < 0.05 was considered to be statistically significant.

## 3 Results

### 3.1 Analysis of the pattern of staining of cerebrovascular basement membrane components within the parenchyma

**Fig. 1:**
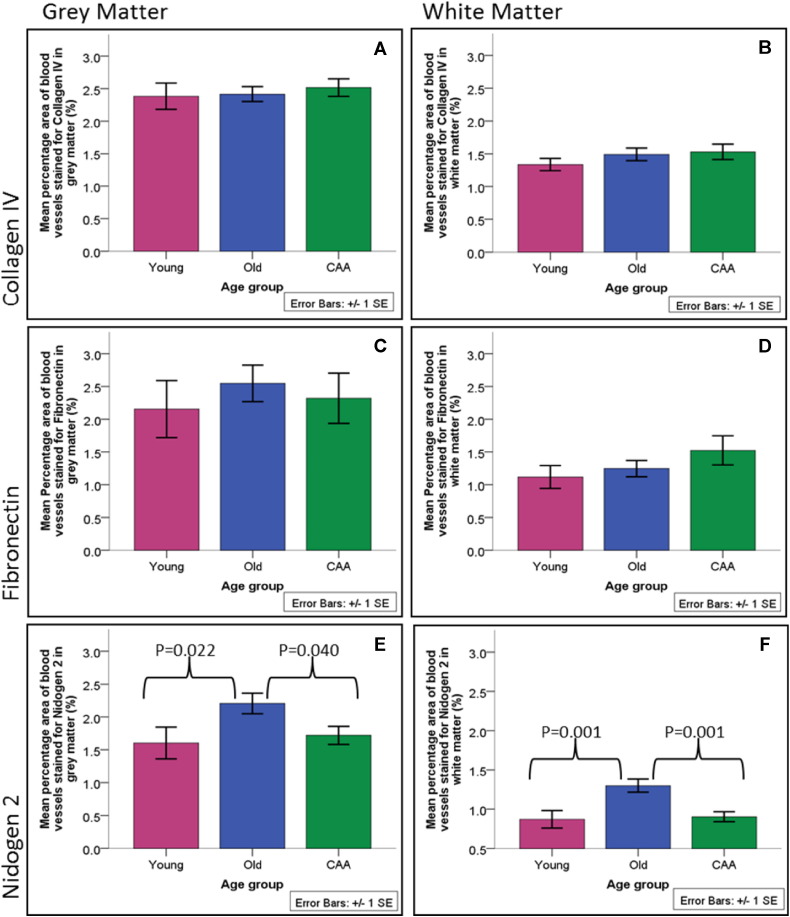
Quantification for collagen IV, fibronectin, nidogen 2 in the gray and white matter of young, old and CAA cases. The percentage areas stained with nidogen 2 in gray and white matter were significantly higher in old compared to young brains and significantly lower in CAA brains compared to old brains.

No significant changes were observed in young, old and CAA brains in the percentage area stained for the collagen IV and fibronectin in the gray or white matter (Fig. 1). The percentage area of the vascular profiles stained with nidogen 2 was significantly higher in old compared to young brains and significantly lower in CAA brains compared to old brains in both gray and white matter (Fig. 1).

### 3.2 Analysis of the main constituents of leptomeningeal artery walls

**Fig. 2:**
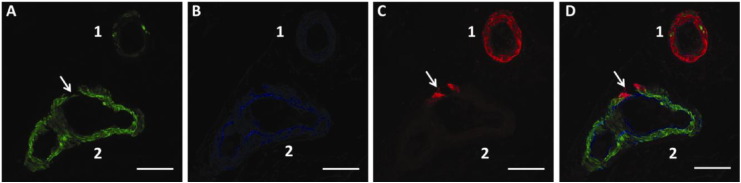
Single colour channel images in series (A–C) and the overlay (D) showing triple immunofluorescence labelling of two leptomeningeal arteries from a severe CAA brain. A) Smooth muscle actin (SMA); B) collagen IV; C) Aβ; D) overlay. Vessel 1 diameter = 71.866 μm, vessel 2 diameter = 80.151 μm. (→) marks a key region of interest where initial Aβ deposition correlates to the only region of reduced SMA in vessel 2. Scale bar: 50 μm.

A fluorescent immunohistochemistry assay was carried out on 10 severe CAA brain tissue sections from Newcastle Brain Tissue Resource and 100 leptomeningeal arteries (10 from each tissue section) were imaged using a Leica SP5 scanning confocal microscope. The pattern of Aβ deposition relative to SMA and Col IV within the leptomeningeal artery walls was qualitatively analysed using single fluorescence channel images in series. This provided a clear image of the pattern of Aβ deposition relative to the vessel wall area and the relative positions of each protein within the vessel morphology. Collagen IV represents the BM and displayed similar morphology and thickness in the basement membranes of the endothelium, tunica media and glia limitans. The presence and morphology of Col IV appeared unchanged between the different severe AD brains, regardless of the Aβ accumulation. The presence of SMA between individual vessels varied, from normal SMA immunostaining within the vessel wall, to minimal/no SMA immunostaining (Fig. 2).

The pattern of Aβ deposition within the leptomeningeal arteries of severe CAA varied greatly, with differing patterns of deposition and degrees of accumulation, from Aβ deposits fully surrounding the perimeter of the vessel wall shown in Fig. 2C vessel 1, to minimal Aβ accumulation in Fig. 2C vessel 2.

### 3.3 Qualitative analysis of co-localization

**Fig. 3:**
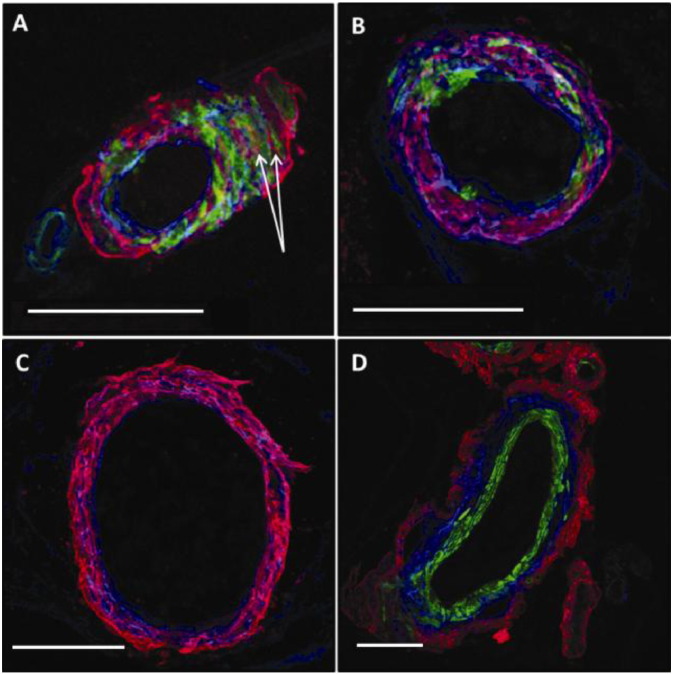
The spectrum of different patterns of deposition of Aβ in CAA. Maximal projection overlaid confocal images of a human leptomeningeal artery from severe CAA brain: A) the basement membrane (collagen IV, blue) of the tunica media is interposed between the smooth muscle cells (green immunolabelling). Aβ (red, arrows) is observed within the basement membranes of tunica media, with blue immunolabelling for collagen IV on both sides; the endothelial BM is free of Aβ; B) co-localization (pink) between the red Aβ and blue Col IV within the BM with the absence of SMA immunolabelling for more of half of the circumference of the arterial wall; C) complete loss of SMA immunolabelling, with Aβ co-localizing with collagen IV in tunica media; D) transverse section through an artery, with Aβ deposited in the adventitia. Blue = Col IV, green = SMA, red = Aβ. Scale bars: 50 μm.

In order to qualitatively analyse the patterns of co-localization between Col IV, SMA and Aβ within the human leptomeningeal artery walls of severe CAA brain tissue sections, maximal projection overlay images were obtained using a Leica SP5 scanning CM. These images comprised of a series of Z-slide images stacked together with all three colour channels: blue for Col IV, green for SMA and red for Aβ, overlaid to show their relative positions within the vessel wall morphology. Qualitative analysis identified regions of red-blue co-localization within a number of the vessels imaged, indicated by the colour purple. We identified a few distinct patterns of deposition of Aβ in the basement membranes: Fig. 3A provides a representation of the visible co-localization (pink) of Col IV (blue) with Aβ (red) within the leptomeningeal artery wall. The Aβ (red) was observed in the BM in tunica media, leaving most of the endothelial and glia limitans BM free (Fig. 3A,B). The basement membranes around smooth muscle cells were occupied by Aβ in a uniform manner for most of their surface (Fig. 3A). Smooth muscle cells were preserved or replaced by Aβ in focal parts of the wall of the artery (Fig. 3B) or entirely (Fig. 3C). Aβ was deposited within the basement membranes of tunica media, with the smooth muscle actin staining intact (Fig. 3A). We observed a distinct pattern of deposition of Aβ on the abluminal aspect of smooth muscle staining, with no immunostaining for basement membranes of the endothelium (Fig. 3D). In the absence of immunostaining for smooth muscle actin, an artery laden with Aβ was identified based on the pattern of deposition of Aβ occupying the entire thickness of the wall, in a lattice pattern. In all images there were 1–3 veins identified based on the pattern of deposition of Aβ, always on the abluminal side of basement membranes and not occupying the entire thickness of the wall.

Quantitative analysis was performed on 100 leptomeningeal arteries from severe CAA brain tissue sections using Image J version Fiji windows 64 software (http://fiji.sc/Fiji; NIH, Bethesda, USA), to analyse:

1. the percentage of the vessel wall occupied by Col IV, SMA and Aβ,
2. the internal diameter of each blood vessel.
3. the degree of co-localization between Col IV, SMA and Aβ in relation to the blood vessel diameter.

**Fig. 4:**
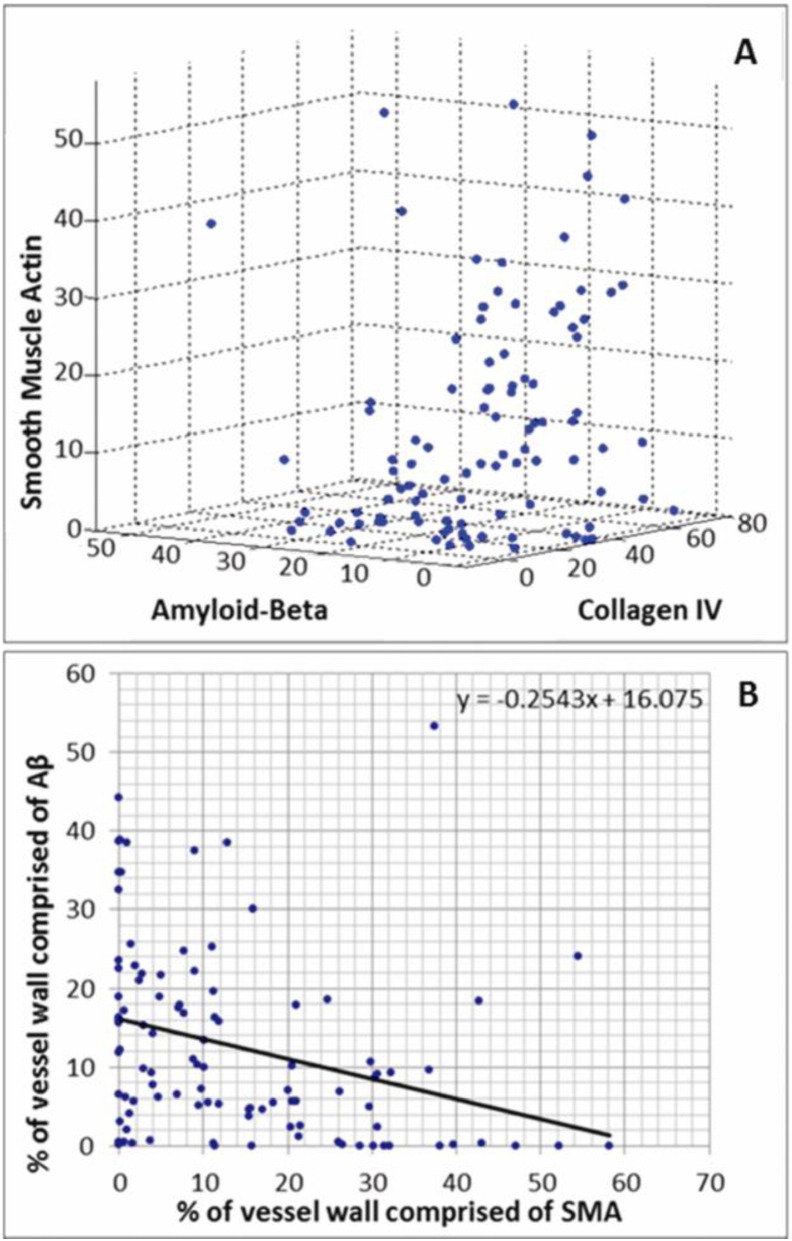
A) The percentage of fluorescently labelled leptomeningeal artery wall comprised of smooth muscle actin, collagen IV and Aβ. B) the relationship between the percentage of fluorescently labelled leptomeningeal artery wall comprised of Aβ and SMA. Each data point corresponds to the values from an individual leptomeningeal artery from the occipital sulcus of a severe CAA brain, 100 vessels in total.

There was a correlation between the amount of Col IV present and amount of Aβ: an increase in Col IV was matched by an increase in Aβ (Fig. 4A). The relationship between SMA and Aβ however shows a general negative correlation: an increase in Aβ was associated with a decrease in the amount of SMA present shown by the negative gradient of the trend line (m = − 0.2543) in Fig. 4B.

**Fig. 5:**
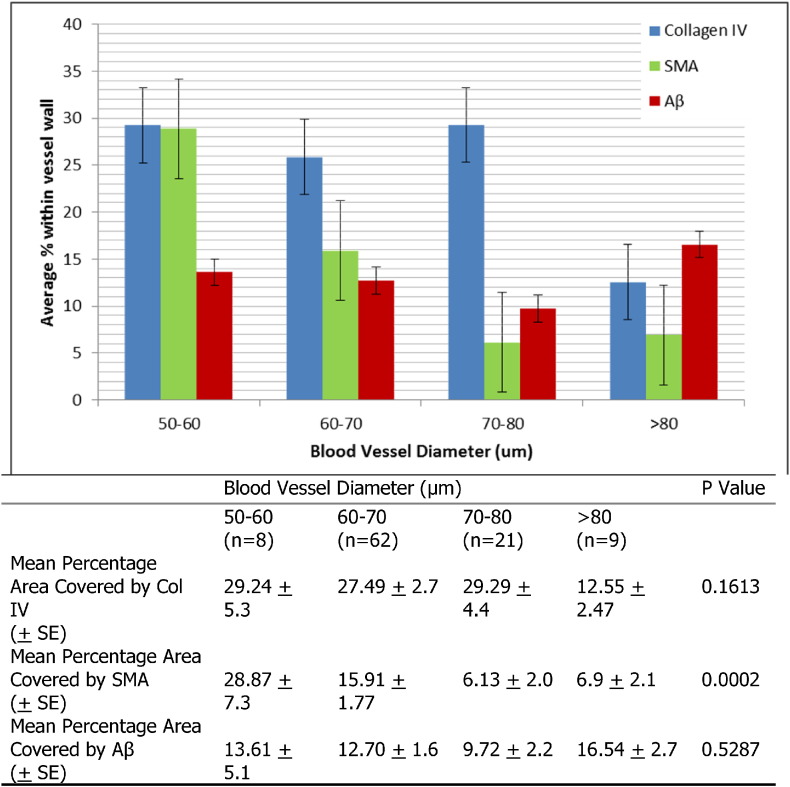
The relationship between blood vessel diameter and expression of SMA, Col IV and Aβ within human leptomeningeal arteries of severe AD brain tissue sections. The average percentage represents the percentage of total fluorescently labelled vessel wall comprised of each protein from an average of ‘n’ vessels. For cohort 50–60 n = 8, 60–70 n = 62, 70–80 n = 21 and > 80 n = 9. The error bars shown represent the standard error. Table: Statistical analysis of the relationship between amount of Col IV, SMA and Aβ expressed in a leptomeningeal artery wall relative to the blood vessel diameter, analysed using Graph Pad Prism 6.0 one-way ANOVA analysis. The percentage area covered by SMA increases with the diameter of the vessel; the percentage area covered by Aβ also increases with the diameter of the vessel, although this did not reach statistical significance. SE: standard error of the mean.

The smaller blood vessels have the least Aβ deposition, correlating to the most SMA and Col IV, whilst the largest blood vessels have the most Aβ deposition, least Col IV and significantly reduced percentage area stained for SMA (p = 0.02) (Fig. 5). The co-localization of collagen IV, SMA and Aβ was calculated using Image J version Fiji windows 64 software (http://fiji.sc/Fiji; NIH, Bethesda, USA) with a two colour channel threshold set in pairs; blue/green, blue/red, red/green. Blue represents Col IV, green represents SMA and red the Aβ. The data from 100 fluorescently labelled leptomeningeal arteries was analysed using Microsoft Excel (Microsoft Office 2010) to find the average degree of co-localization between the three pairings from all 100 vessels. The highest degree of co-localization was observed between Col IV and Aβ, within leptomeningeal arteries of severe CAA brains (Table 4).

## 4 Discussion

Experimental studies suggest that lymphatic drainage of fluid and solutes from the brain occurs along basement membranes of capillaries and arteries and that such drainage is impaired by age and CAA [14], [17]. Here we propose a sequence of changes whereby Aβ is initially deposited in the basement membranes surrounding smooth muscle cells. There are distinct patterns suggesting progression from Aβ deposition in the central part of basement membranes, to complete co-localization of Aβ with basement membranes in tunica media, leaving the endothelial basement membranes free. Nidogen (entactin) prevents the aggregation of Aβ (28). We observed a significant increase in the amount of nidogen (entactin) with normal ageing, possibly indicating a compensatory mechanism for the prevention of aggregation of Aβ in the vascular walls.

**Fig. 6:**
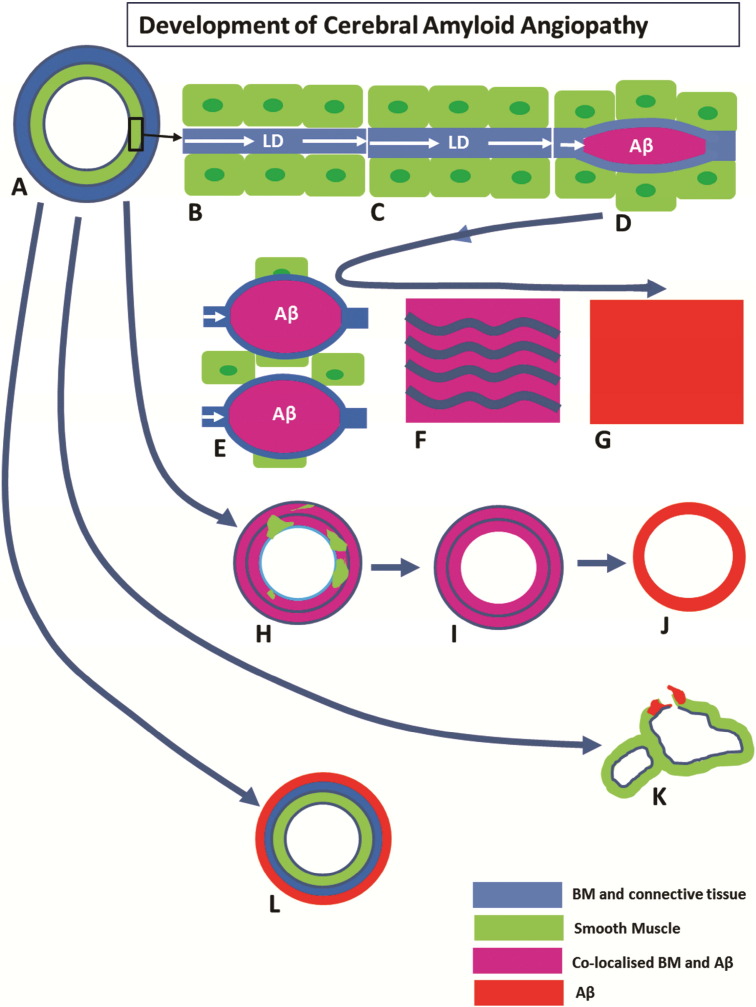
Development of cerebral amyloid angiopathy.

We have identified a number of stages through which this sequence passes (Fig. 6) from the initial deposition of Aβ within basement membranes between smooth muscle cells walls of the artery to complete replacement of the wall by Aβ. Previous ultrastructural studies have reported the progressive deposition of fibrillar amyloid in the lamina densa of smooth muscle basement membranes in the tunica media of leptomeningeal arteries in the development of CAA [29], [30]. The authors at that time suggested that the sole source of Aβ was smooth muscle cells. It is now clear that basement membranes form the pathway by which Aβ drains from the brain and that the largest proportion of Aβ in smooth muscle basement membranes in CAA is derived from the brain [31], [32]. As the volume of an Aβ deposit increases in size, it separates the smooth muscle basement membrane into its two component parts as shown here and previously (21). With further growth in size of Aβ deposits, smooth muscle cells are lost from the tunica media, possibly associated with destruction of their basement membranes. As observed by the co-localization of Aβ with the basement membrane protein collagen IV, there is some preservation of basement membrane elements within the artery wall, even after the loss of smooth muscle cells. Eventually, all basement membrane elements are lost and the vessel wall is composed solely of Aβ.

There are variations in the sequence of stages described above and these are also shown in Fig. 6. In some arteries, deposition of Aβ remains focal, even to the point of complete replacement of smooth muscle and basement membrane elements by Aβ. The apparent rupture of the vessel in Fig. 2C,D cannot be ascribed with certainty to an in vivo event in this case, but it shows a potential site of weakness in the vessel wall that may in some cases be associated with rupture and intracerebral haemorrhage. The heterogeneity in presentations may be due to how different risk factors for CAA affect the process of perivascular clearance. For example, possession of ApoE4 genotype alters the biochemical composition of basement membranes, whereas mid-life hypertension alters the biophysical forces acting upon the arterial wall, modifying the motive force for perivascular clearance. Another feature is depicted in Fig. 3D, in which the tunica media is completely free of Aβ and there is preservation of the smooth muscle cells. Instead, Aβ is deposited in the tunica adventitia, which may represent part of the lymphatic drainage pathway to lymph nodes in the neck, but this exact route still requires strict verification.

(a) A normal leptomeningeal artery showing the tunica media composed of smooth muscle cells (green) and connective tissue of the tunica adventitia (blue). (b) Tunica media showing smooth muscle cells (green) and intervening basement membrane (blue). Lymphatic drainage (LD) of interstitial fluid and solutes, including soluble Aβ, from the brain occurs along basement membranes (BM) in the tunica media of cerebral arteries. (c)–(g) depict the age-related thickening of arterial BM (c) with impaired LD, through the stage of amyloid co-localisation with basement membranes (d) (see Fig. 3A), to replacement of smooth muscle cells by Aβ (e–g), with some preservation of basement membrane material (f) to complete replacement of the artery wall by Aβ (g). Figures (h–j) (see Fig. 3B–C) show patterns of replacement of artery wall by Aβ. Most of the smooth muscle cells are replaced with Aβ co-localized with basement membrane material (h) to complete loss of smooth muscle cells but with some preservation of basement membrane (i). Finally the whole vessel wall is replaced by Aβ (j) (see vessel 1 in Fig. 2). Variations in the pattern of deposition of Aβ are seen in figures (k)–(l). Focal complete replacement of vessel wall by Aβ is seen in (k) (see vessel 2 in Fig. 2). Deposition of Aβ in the tunica adventitia in (l) (see Fig. 3d) may represent part of the lymphatic drainage pathway of Aβ along the adventitia of leptomeningeal arteries.

### 4.1 Relationship of perivascular drainage of Aβ to other pathways of Aβ elimination

A number of pathways for the elimination of Aβ from the brain have been identified and they include receptor-mediated absorption of Aβ into the blood (33), degradation of Aβ by enzymes such as neprilysin (34), drainage of Aβ into the CSF (35) as well as perivascular lymphatic drainage (14). Impairment of both neprilysin and absorption of Aβ into the blood appear to result in increased severity of CAA, suggesting that Aβ is diverted to perivascular drainage pathways [36], [37]. Although there is physiological evidence that Aβ introduced into the CSF passes into the interstitial fluid of the brain and thence returns to the CSF (38), the data are derived from animal experiments and that there are no data from human studies to show that Aβ is deposited in these pathways in Alzheimer's disease.

### 4.2 Consequences of age-related changes in cerebral arteries and CAA

As arteries age, they become stiffer, with loss of innervation and increasing rigidity that is associated with impairment of perivascular drainage of fluid and solutes [39], [40], [41]. It appears that such impairment may not only result in CAA, but also act as a trigger for loss of homoeostasis in the brain, a rise in soluble Aβ and seeding of Aβ plaques in brain parenchyma. Furthermore, age-related changes may initiate the amyloid cascade that results in neuronal damage and acceleration of tau propagation in the pathogenesis of Alzheimer's disease (42).

The other major complication of CAA is intracerebral haemorrhage, although why such haemorrhages show spatial clustering and tend to involve the temporal and occipital lobes preferentially is unclear (43). The present study shows how amyloid expanding from the basement membrane drainage pathways may eventually totally replace all elements of an artery wall. Further factors that result in rupture of vessels associated with CAA are not clear. The degree of replacement of the vessel wall by amyloid required before the vessel ruptures is at the moment unknown. Further study of arteries associated with intracerebral haemorrhage may help to answer this question.

### 4.3 Conclusion

In CAA, Aβ is deposited within the perivascular drainage pathways of the brain. The cerebrovascular basement membranes undergo biochemical changes with increasing age and there are specific patterns of vascular morphology associated with ageing. The morphological patterns of vascular anatomy may become future markers for the efficiency of perivascular drainage and the risk of CAA.

## Author contributions

Roxana O Carare designed the study. Abby Keable performed the quantitative iimmunohistochemistry and optimized antibodies. Kate Fenna and David Johnston performed the confocal microscopy. Ho Ming Yuen performed the statistical analysis. Colin Smith, Rustam Al-Shahi Salman, Neshika Samarasekera, Johannes Attems diagnosed the cases and provided the tissue. James Nicoll, Raj Kalaria and Roy O Weller assisted with the design of the study, analysis and drafting of the manuscript. Neil Smyth produced and provided the anti-nidogen 2 antibody.

## Conflict of interests

All authors declare no conflict of interest for this manuscript

## Transparency document

Transparency document.

## Figures and Tables

**Table 1:** a). Demographics of cases used in this study. Brains from young cases were from the MRC Sudden Death Brain& Tissue Bank (Edinburgh). Brains from old non-demented and CAA cases were from Newcastle Brain Tissue Resource; b). diagnoses for the cases assessed.

a)								
Young			Aged non-demented controls			CAA		
Case ID	Age	Gender	Case ID	Age	Gender	Case ID	Age	Gender
SD020 12L	56	M	4309L	96	F	2609AM	83	M
SD023 12L	50	M	6709L	74	F	5109L	79	F
SD032 11M	58	M	8108L	79	F	6509L	75	M
SD033 10N	51	M	8210L	72	M	7709L	63	F
SD036 10N	21	M	10,908L	81	M	8809L	88	F
SD039 10N	48	M	32,212L	90	M	9810L	86	F
SD042 12M	29	M	32,412L	50	M	10,009L	80	F
SD045 12M	37	M	34,012L	95	M	10,208L	84	F
UA09 424	50	M	35,310L	74	F	10,409L	79	M
UA09 527	46	M	35,910L	94	F	31,011L	91	F
UA09 588	51	M	47,711L	95	F	32,010L	88	M
UA09 611	56	M	48,612L	92	F	42,610L	84	F
UA09 633	49	M	52,411L	83	F	50,510L	86	F
UA09 634	32	M	57,510L	77	M	55,710L	85	M
UA09 644	44	F	64,811L	89	F	71,511L	83	M
UA10 23	38	M	68,510L	88	F	72,510L	87	F
UA10 210	36	M	72,910L	70	M	98,710L	81	F
UA10 222	27	M	73,611L	81	F	102,610L	93	F
UA10 319	43	M	89,111L	73	M	104,010L	78	M
SD024 12M	44	M	113,511L	76	F	111,510L	77	M
Age range	(21–58) = 37			(50–96) = 46			(63–93) = 30	
Mean	43.3			81.45			82.5	
Standard deviation	10.065			11.115			6.399	


b)			
Case ID	Group	AD Braak	Diagnosis
4309L	Old	2	Clinical dementia — no pathology
6709L	Old	1	Cognitively normal control
8108L	Old	4	Parkinson's disease with DLB
8210L	Old	0	Frontotemporal dementia
10,908L	Old	3	Parkinson's disease without dementia
32,212L	Old	2	Parkinsons disease without dementia
32,412L	Old	0	Corticobasal degeneration (but not cognitively impaired)
34,012L	Old	3	Alzheimer's disease
35,310L	Old	3	Cognitively normal control
35,910L	Old	2	Cognitively normal control
47,711L	Old	3	Cognitively normal control
48,612L	Old	2	Cognitively normal control
52,411L	Old	1	Major depression, no dementia
57,510L	Old	3	Dementia with Lewy bodies (DLB)
64,811L	Old	3	Cognitively normal control
68,510L	Old	3	Cognitively normal control
72,910L	Old	0	Cognitively normal control
73,611L	Old	3	Progressive supranuclear palsy
89,111L	Old	0	Cognitively normal control
113,511L	Old	2	Parkinson's disease with non-DLB dementia
2609AM	CAA	6	Alzheimer's disease
5109L	CAA	4	Frontotemporal dementia
6509L	CAA	4	Alzheimer's disease
7709L	CAA	6	Mixed type dementia: Alzheimer's disease and DLB
8809L	CAA	2	Alzheimer's disease
9810L	CAA	6	Alzheimer's disease
10,009L	CAA	6	Alzheimer's disease
10,208L	CAA	6	Alzheimer's disease
10,409L	CAA	3	Stroke without dementia
31,011L	CAA	3	Dementia with Lewy bodies
32,010L	CAA	6	Alzheimer's disease
42,610L	CAA	6	Alzheimer's disease
50,510L	CAA	6	Alzheimer's disease
55,710L	CAA	6	Alzheimer's disease
71,511L	CAA	4	Parkinson's disease with DLB
72,510L	CAA	6	Alzheimer's disease
98,710L	CAA	6	Alzheimer's disease
102,610L	CAA	5	Alzheimer's disease
104,010L	CAA	6	Alzheimer's disease in advanced isocortical stage with mild CAA type 2 and DLB
111,510L	CAA	6	Alzheimer's disease in advanced isocortical stage with moderate CAA type 2 and DLB

**Table 2:** Number of cases and images from each group: a) total number of cases; b) total number of images from each group examined.

a)			
Marker	Group		
	Young	Old	CAA
Collagen IV	19	19	20
Fibronectin	18	20	19
Nidogen 2	14	20	20


b)									
Marker	Group								
	Young			Old			CAA		
	Grey	White	Total	Grey	White	Total	Grey	White	Total
Collagen IV	94	95	189	95	95	190	100	100	200
Fibronectin	90	90	180	100	100	200	95	95	190
Nidogen 2	70	70	140	100	100	200	100	100	200

**Table 3:** Details of the primary and secondary antibodies used for immunohistochemistry staining.

Antibody type	Antigen	Name provided by supplier	Supplier details	Dilution
Primary	Collagen IV	Rabbit anti-collagen IV	Anti-collagen IV primary antibody, polyclonal, produced in Rabbit (ab6586) AbCam, Cambridge, UK	1:400
Primary	Smooth muscle actin	FITC conjugated mouse anti-alpha chain SMA	Anti-alpha chain SMA primary monoclonal antibody (F3777) Sigma Aldrich, Dorset, UK	1:200
Primary	Aβ	Mouse anti-Aβ IgG2b SIG39220	Anti Aβ 17–24 (4G8) primary monoclonal antibody (SIG39220) Covance, Cambridge Bioscience, Cambridge, UK	1:100
Secondary	Rabbit IgG	Alexa fluor 633 goat anti-rabbit	Alexa fluor 633 goat anti-rabbit IgG, polyclonal, (A-21,071) Invitrogen, Life Technologies, Paisley, UK	1:200
Secondary	Mouse IgG2b	Alexa fluor 594 IgG2b goat anti-mouse	Alexa fluor 594 goat anti-mouse IgG2b (A-21,145) Invitrogen, Life Technologies, Paisley, UK	1:200

**Table 4:** Statistical analysis of the differences in amount of co-localization measured between each protein pair (Col IV/SMA, Col IV/Aβ and SMA/Aβ) in leptomeningeal artery walls of severe CAA brains, analysed using Graph Pad Prism 6.0 two-way ANOVA analysis. There is significantly more co-localization between collagen IV and Aβ compared to SMA and Aβ.

Co-localization protein pairing	% of total variation	P value
Col IV/SMA vs Col IV/Aβ	57.87	0.0262
Col IV/SMA vs Aβ/SMA	67.65	< 0.0001
Col IV/Aβ vs Aβ/SMA	54.85	0.0050
